# Emerging Role of Kinin B1 Receptor in Persistent Neuroinflammation and Neuropsychiatric Symptoms in Mice Following Recovery from SARS-CoV-2 Infection

**DOI:** 10.3390/cells12162107

**Published:** 2023-08-19

**Authors:** Srinivas Sriramula, Drew Theobald, Rohan Umesh Parekh, Shaw M. Akula, Dorcas P. O’Rourke, Jeffrey B. Eells

**Affiliations:** 1 Department of Pharmacology and Toxicology, Brody School of Medicine, East Carolina University, Greenville, NC 27834, USA; theobaldd21@students.ecu.edu (D.T.); parekhr13@students.ecu.edu (R.U.P.); 2 Department of Microbiology and Immunology, Brody School of Medicine, East Carolina University, Greenville, NC 27834, USA; akulas@ecu.edu; 3 Department of Comparative Medicine, Brody School of Medicine, East Carolina University, Greenville, NC 27834, USA; orourked@ecu.edu; 4 Department of Anatomy and Cell Biology, Brody School of Medicine, East Carolina University, Greenville, NC 27834, USA

**Keywords:** kinin B1 receptor, neuroinflammation, SARS-CoV-2, COVID-19, K18-hACE2 mice, long COVID

## Abstract

Evidence suggests that patients with long COVID can experience neuropsychiatric, neurologic, and cognitive symptoms. However, these clinical data are mostly associational studies complicated by confounding variables, thus the mechanisms responsible for persistent symptoms are unknown. Here we establish an animal model of long-lasting effects on the brain by eliciting mild disease in K18-hACE2 mice. Male and female K18-hACE2 mice were infected with 4 × 10^3^ TCID50 of SARS-CoV-2 and, following recovery from acute infection, were tested in the open field, zero maze, and Y maze, starting 30 days post infection. Following recovery from SARS-CoV-2 infection, K18-hACE2 mice showed the characteristic lung fibrosis associated with SARS-CoV-2 infection, which correlates with increased expression of the pro-inflammatory kinin B1 receptor (B1R). These mice also had elevated expression of B1R and inflammatory markers in the brain and exhibited behavioral alterations such as elevated anxiety and attenuated exploratory behavior. Our data demonstrate that K18-hACE2 mice exhibit persistent effects of SARS-CoV-2 infection on brain tissue, revealing the potential for using this model of high sensitivity to SARS-CoV-2 to investigate mechanisms contributing to long COVID symptoms in at-risk populations. These results further suggest that elevated B1R expression may drive the long-lasting inflammatory response associated with SARS-CoV-2 infection.

## 1. Introduction

The novel severe acute respiratory syndrome coronavirus 2 (SARS-CoV-2) was first identified at the end of 2019 and is responsible for the coronavirus disease 2019 (COVID-19) pandemic. Classic cases of acute COVID-19 are characterized by lower respiratory tract symptoms, fever, and gastrointestinal problems [1]. However, clinical manifestations of patients infected with SARS-CoV-2 vary in severity from asymptomatic to acute respiratory distress syndrome, multiple organ failure, and cardiovascular, renal, and neurological complications that can lead to death. The primary consequence of SARS-CoV-2 infection is an inflammatory response in multiple organ systems, leading to cytokine and bradykinin storms, which are considered the cause of pathological manifestations of COVID-19 [2,3,4].

A growing concern is that patients infected with SARS-CoV-2 report persistent symptoms that do not resolve over the course of many months. These patients’ symptoms have been termed long COVID, post-acute COVID-19 syndrome (PACS), or post-acute sequelae of SARS-CoV-2 infection (PASC) [5,6]. The Centers for Disease Control and Prevention defines long COVID as symptoms that persist for more than a month after infection with SARS-CoV-2. Currently, this definition includes infected individuals ranging from asymptomatic or mild-to-moderate symptoms to severe disease requiring hospitalization. A recent study reported that 33% of patients experienced neurological or neuropsychiatric symptoms 6 months after recovery from COVID-19 [7]. Their persistent neurological symptoms included fatigue, depression, anxiety, cognitive problems, brain fog, and sleep disturbances, even in individuals who experienced only mild symptoms [8]. Such persistent neurological and neuropsychiatric symptoms [8] can contribute to neurodegenerative diseases such as Alzheimer’s disease (AD) and dementia [9], as has been suggested for other viral infections [10,11,12]. As a result of advances in treatment and development of vaccines, the number of patients recovering from COVID-19 is rising; therefore, millions of people globally could be susceptible to long-term neurological, neuropsychiatric, and neurodegenerative complications [10,11,12]. However, there is no clear understanding of the relationship between the SARS-CoV-2 infection and long-term complications, as clinical data from patients infected with SARS-CoV-2 are mostly associational studies confounded by multiple variables. 

There is evidence that SARS-CoV-2 has the capability to invade neural tissue. Brain imaging studies have revealed hypometabolism, functional changes, and inflammation in the brains of patients with long COVID [13]. Several mechanisms have been proposed to account for these persistent neurological symptoms, including vascular dysfunction, peripheral inflammation, and autoantibodies, as well as direct infection of the brain (via active replication or limited replication with an immune response) [14]. Previous studies show that SARS-CoV-2 can enter the central nervous system in humans [15,16,17]. Additionally, the virus or antibodies to SARS-CoV-2 in the cerebrospinal fluid (CSF) during COVID-19 have been detected, but not in all cases [15,16]. Human brain organoids have provided additional evidence for the ability of SARS-CoV-2 to infect human neurons [18]. Therefore, the role of neurotropism of SARS-CoV-2 is unclear and the mechanisms responsible for its chronic effects on the brain are yet to be defined.

SARS-CoV-2 infects cells primarily by binding to angiotensin-converting enzyme 2 (ACE2), which ultimately diminishes the function of this receptor. Furthermore, ACE2 can specifically inactivate the active metabolite of bradykinin, des-Arg^9^-bradykinin (DABK), which is a specific inducer of kinin B1 receptor (B1R) expression. Once B1Rs are induced, they cannot be internalized or desensitized, and can drive further inflammation. Evidence suggests that bradykinin homeostasis is disrupted by SARS-CoV-2 infection [19]. Increased levels of bradykinin have been detected in the bronchoalveolar lavage fluid of COVID-19 patients during acute or severe disease [4]. Thus, a possible mechanism driving the long-term effects of SARS-CoV-2 infection is induction of B1R expression. 

We hypothesized that SARS-CoV-2 infection induces long-lasting B1R expression in the brain, driving chronic inflammation and ultimately cognitive and neuropsychiatric symptoms. To test this experimentally, we sought to develop an animal model of long COVID. SARS-CoV-2 infection and COVID-19 have been modeled in different animal species, including hamsters, ferrets, and non-human primates [20,21,22,23,24]. However, most species exhibited only mild disease and not the range of severity—asymptomatic to severe disease and death—that occurs in humans. We utilized transgenic mice expressing human ACE2 under control of the human keratin 18 promoter (K18-hACE2 mice), which are widely used as a laboratory animal model suitable for studying SARS-CoV-2 pathogenesis [23,25,26]. Because these mice are very sensitive to SARS-CoV-2 infection, frequently exhibiting severe disease but surviving lower doses, they are an excellent model of SARS-CoV-2 infection. However, most studies of this mouse model have focused on the acute effects of SARS-CoV-2 infection and severe disease [22,23]. Chronic effects on the brain of K18-hACE2 mice surviving SARS-CoV-2 infection have not been reported. 

To establish a robust model of long COVID, we determined the dose of SARS-CoV-2 in which 80% of K18-hACE2 mice survive. Following recovery from such mild SARS-CoV-2 infection, the lungs of these mice displayed obvious fibrosis and elevated levels of B1R expression. Because the functions of the hippocampus (spatial memory), amygdala (processing fear/anxiety), and prefrontal cortex (motor planning) are associated with cognitive and neuropsychiatric symptoms reported in long COVID, we investigated bradykinin signaling and inflammation in these regions. Elevated expression of B1R and the cytokines interleukin 6 (IL-6) and tumor necrosis factor-alpha (TNF) were observed in these brain regions. Since there is a cross talk between B1R and angiotensin II type 1 receptor (AT1R) in mediating neuroinflammation [27], we also measured the AT1R expression and found that elevated expression of AT1R correlated with increased neuroinflammation. Importantly, these long-term neuronal changes are associated with persistent behavioral changes, including elevated anxiety in the zero maze and altered exploratory behavior in the Y maze. Our data thus demonstrate the potential of SARS-CoV-2 to cause persistent effects on bradykinin signaling and brain function. Because the K18-hACE2 mice are very susceptible to SARS-CoV-2 infection, this initial investigation of the long-term behavioral effects of SARS-CoV-2 infection suggests that these effects could be replicated in humans most at risk for long COVID and contribute to persistent neurological symptoms following infection. Because elevated inflammation has been associated with increased risk of neurodegenerative diseases such as Parkinson’s disease, AD, and other dementias, we suggest that SARS-CoV-2 infection may contribute to an elevated risk for these diseases. Finally, our model could be used to investigate the mechanisms responsible for long COVID following recovery from a mild infection and confirm our proposal that elevated B1R expression is a critical mechanism driving inflammation in the brain following SARS-CoV-2 infection.

## 2. Materials and Methods

### 2.1. Study Approval

Animal experiment approval was provided by the Institutional Animal Care and Use Committee (IACUC) protocol (AUP#A209) at East Carolina University (AAALAC-accredited facility, Animal welfare assurance number A3469–01). The Institutional Biosafety Committee (IBC) approved work with infectious SARS-CoV-2 virus strains under BSL3 conditions in an AAALAC-accredited facility by certified staff, following the basic principles and guidelines in the NIH Guide for the Care and Use of Laboratory Animals. All sample inactivation was performed according to IBC-approved standard operating procedures for removal of specimens from high containment.

### 2.2. SARS-CoV-2 Virus

All the work pertaining to the use of SARS-CoV-2 was performed in a BSL-3 laboratory. SARS-CoV-2 (isolate USA-WA1/2020) was purchased from BEI Resources (Manassas, VA, USA) and was propagated, purified, and titrated in Vero cells as per the standard protocols [28]. 

### 2.3. Experimental Design

Transgenic mice with the human ACE2 gene under the control of K18 promoter (B6.Cg-Tg(K18-ACE2)2Prlmn/J, Jackson Labs, Strain #034860) were used in these studies. Prior to infection, the mice were implanted with a UID Temperature Microchip and continuously monitored for body temperature and activity using the UID Mouse Matrix (UIdevices). Male and female hACE2-K18 mice, ranging in age 6–9 months, were randomly assigned to receive either vehicle saline (mock controls) or SARS-CoV-2 at a dose of 1 × 10^4^ or 4 × 10^3^ 50% tissue culture infectivity dose (TCID50). Mice were anesthetized with 3–4% isoflurane, and 12.5 ml of either the vehicle or SARS-CoV-2 was pipetted into each naris (25 μL total volume). Following infection, mice were housed individually and managed in an ABSL3 facility (East Carolina University, Greenville, NC). Mice were weighed daily and monitored daily for symptoms for 10 days after infection. Mice that had rapid weight loss, lethargy, and became moribund between 6 and 10 days post-infection (dpi) were euthanized. Behavior and cognitive function tests were performed starting from 30 dpi. The mice were euthanized, and tissues were harvested at 45 dpi.

### 2.4. Behavior and Cognitive Function Tests

Behaviors in mice were tested starting 30 dpi. All testing was performed between 1300 and 1600 h. Treatment groups were distributed evenly throughout the testing time. The behavior apparatuses were enclosed in a Duo-Flo™ mobile unit with BioClean™ clean room technology with LED lighting providing approximately 100 lux of illumination. EthoVision software was used to track the movement of the mice. For the open field test, mice were placed in the middle of an open field chamber (Stoelting open field, 40 cm × 40 cm, clear plexiglass) and video was recorded for 10 min. Parameters measured in the open field test included total distance, acceleration, time moving, and time around the border. Open field zones were established as a 10 cm wide zone around the border and a 20 cm × 20 cm center zone. For the anxiety assessment, a Stoelting mouse zero maze was used, 50 cm in diameter with 5 cm lanes and an adjustable rim at 3 mm. The mice were placed inside the closed portion of the maze and recorded for 6 min. For the zero maze test, time spent in the open region was determined using EthoVision software with manual scoring to measure total time in the open (defined as any part of the mouse entering the open portion of the maze), total entries into the open, time with entire body of mouse in the open, and number of whole-body entries. For the Y maze test, a Stoelting Y maze for mice was used with lanes 5 cm wide and with a 35 cm arm length. Mice were placed in one arm and allowed to explore freely for 5 min. Video of each session was recorded. Using the EthoVision software, a zone for each arm was made and a center zone extended 2.5 cm down each arm. An entry into an arm was counted when the center point of the mouse crossed from the center zone into an arm. The number of entries were manually counted with the aid of the software. A spontaneous alternation was defined as entries into three different arms consecutively (i.e., ABC, CAB, or BCA, but not BAB and CBC). The number of total arm entries served as an indicator of exploratory behavior. The velocity, time immobile, and total distance traveled was determined by the tracking software and served as a measure of locomotor activity. All behavioral measurements were made blinded to treatment. Following each test, the mazes were cleaned with 70% isopropyl alcohol to remove residual odors and disinfect for any possible virus shedding.

### 2.5. Histology and Immunohistochemistry

Mice that developed severe disease were deeply anesthetized with 5% isoflurane, euthanized by decapitation, and the brain and lungs were fixed in 10% formalin. For transcardial perfusion, mice were deeply anesthetized and maintained on 5% isoflurane, the thoracic cavity was opened, and a blood sample was collected via cardiac puncture. Mice were then transcardially perfused with phosphate buffered saline followed by 3% paraformaldehyde. Brain and lung tissue was collected and post-fixed in 3% paraformaldehyde. Lung tissue and olfactory bulb tissue was dehydrated in 70% ethanol, embedded in paraffin, and sectioned at 5 μm. Lung and olfactory bulb sections from severe disease mice were processed for SARS-CoV-2 (#40150-MM150, lot HB14AP0201-B, Sino Biological, PA, USA, 1:1000) immunohistochemistry. Lung sections from mild disease mice were processed for Masson’s trichrome staining and immunohistochemistry. B1R-specific antibody B1R (#ABR-011, lot An-01, Alomone labs, Jerusalem, Israel; 1:500 dilution) and SARS-CoV-2 Spike S1 subunit (#40150-MM10, lot HB14AP0201-B, Sino Biological, Wayne, PA, USA., 1:1000) were used for immunohistochemical staining. The sections were incubated with the primary antibodies overnight at 4 °C and developed using ImmPRESS Excel Staining Kit (MP-7601, Vector Laboratories, Newark, CA, USA) following the manufacturer’s instructions. All slides were imaged using a Philips IntelliSite Ultra-Fast Slide Scanner. Representative images were then extracted and shown. Quantification of the collagen deposition and B1R immunoreactivity was performed using NIH ImageJ software and presented as percent area of staining. For Iba-1 immunostaining, sections were incubated with anti-goat biotinylated antibody followed by avidin-biotinylated-HRP complex (Vector Laboratories, Newark, CA, USA). Immunostaining was developed using the ImmPACT DAB solution for 4 min, washed and mounted on silanized slides, dehydrated with graded ethanol, defatted in xylene, and cover slipped using Permount. To quantify microglia reactivity, Iba1 labeled images of the hippocampus, amygdala, and prefrontal cortex were acquired at 40× magnification on the MoticEasyScan slide scanner using the extended depth of focus feature. An area of the image (615 μm × 300 μm) was captured and used to quantify microglia activation. Microglia cell bodies were counted in this area. The distribution of Iba-1 staining was quantified using ImageJ. Microglia activation was determined based on the percent area of Iba-1 staining and reported relative to the number of microglia cell bodies. A total of 4–6 images for each region was used to quantify microglia activity. To measure the area of the microglia cell body, the images were processed as described previously [29,30]. Briefly, the images were enhanced using the default FFT-Bandpass process then converted to gray scale. The brightness/contrast was adjusted then the unsharp mask filter was used. The rectangular selection tool was used to randomly select 10–12 cells using a grid pattern to randomly choose cells. The paint brush tool was used to remove processes from cells. The area of each cell was then measured. To further investigate changes in microglia morphology, the complexity in branching of microglia processes was measured (number of branches, number of junctions, number of end points) with the Skeleton 2D/3D plugin in ImageJ. The cell body and the branches of each microglia in a randomly selected image (between 30 and 40 cells) were traced, converted to binary, skeletonized, and analyzed with ImageJ. 

For the immunofluorescence staining protocol, following fixation, brain tissue was cryoprotected in a 30% sucrose solution for 24 h. Then a cryostat was used to cut 30 μm free-floating sections. Free-floating brain sections were washed with PBS and blocked with 5% donkey serum in 1 × PBS containing 0.2% Tween-20 for 1 h. They were then incubated with appropriate primary antibodies: B1R (#ABR-011, lot An-01, Alomone labs, Jerusalem, Israel; 1:500 dilution), IL-6 (12912S, lot 2, Cell Signaling Technologies, Beverly, MA, USA; 1:500) TNF (11948S, lot 5, Cell Signaling Technologies, Beverly, MA, USA; 1:500), AT1R (#AAR-011, lot AAR011AN2002, Alomone labs, Jerusalem, Israel; 1:500), NeuN (MAB377, lot 3808682, Millipore Sigma, Burlington, MA, USA; 1:500), SARS-CoV antiserum (NR-10362, BEI resources, Manassas, VA, USA; 1:500), and dsRNA (Ab01299-23.0, absolute antibody, Boston, MA, USA; 1:500). The next day, sections were washed with PBS + 0.2% Tween-20 and incubated with appropriate Alexa flour conjugated secondary antibodies (ThermoFisher, Hanover Park, IL, USA; 1:1000 dilution) for 1 h at room temperature, followed by DAPI nuclear stain. Sections were mounted with ProLong Diamond Anti-Fade Mount (ThermoFisher, Hanover Park, IL, USA). Images were captured using an Echo Revolve or Keyence Microscope. We used previously validated primary and secondary antibodies for immunostaining, and no primary antibody negative controls were included in every experiment to assess the background fluorescence caused by non-specific binding of the antibody [27,31,32]. All experiments and data analyses were performed independently in a blinded fashion by at least two independent investigators.

### 2.6. RNA Extraction and Real-Time Quantitative RT-PCR

Hippocampus and prefrontal cortex tissue samples were microdissected and total RNA was extracted from individual tissue samples using a Quick-RNA FFPE miniprep kit (Zymo Research, Irvine, CA, USA). RNA concentration and purity were assessed using a spectrophotometer (NanoDrop One, ThermoFisher, Hanover Park, IL, USA). Real-time quantitative PCR (RT-qPCR) was performed with PowerUP SYBR green master mix (ThermoFisher, Hanover Park, IL, USA) using primer pairs listed in Supplementary Table S1. Each reaction was performed in triplicate. Data were normalized to β-actin expression by the 2^−(ΔΔCT)^ comparative method and expressed as a fold change compared to control.

### 2.7. SARS-CoV-2 Antibody Titer Measurement Using ELISA

Blood samples collected via cardiac puncture prior to perfusion were placed in KE EDTA tubes and plasma was isolated. Antibody titers were measured in plasma using the Mouse Anti-SARS-CoV-2 IgG Antibody ELISA Kit (DEIASL240, Creative Diagnostics, Shirley, NY, USA) according to the manufacturer’s instructions. Initial concentrations were measured using 1 ml of plasma. A second measurement was determined with either a 1:10 or 1:20 dilution of plasma, based on the initial screening, to obtain measurements within the linear range of the standard curve.

### 2.8. Statistical Analysis

Statistical significance was determined using GraphPad Prism 10.0.1 (GraphPad Software, Boston, MA, USA). The normality of the data was tested (Shapiro–Wilk test), and appropriate methods were chosen for comparative statistics. Data are presented as mean ± standard error of the mean (s.e.m.). For the comparison of 2 independent data sets, an unpaired, 2-tailed Student’s *t* or Mann–Whitney U test was used for normally and non-normally distributed data, respectively. The correlation analysis of B1R expression and fibrosis was performed using linear regression analysis. Differences were considered statistically significant at *p* < 0.05.

## 3. Results

### 3.1. K18-hACE2 Mice Are Susceptible to Severe SARS-CoV-2 Infection 

**Figure 1:**
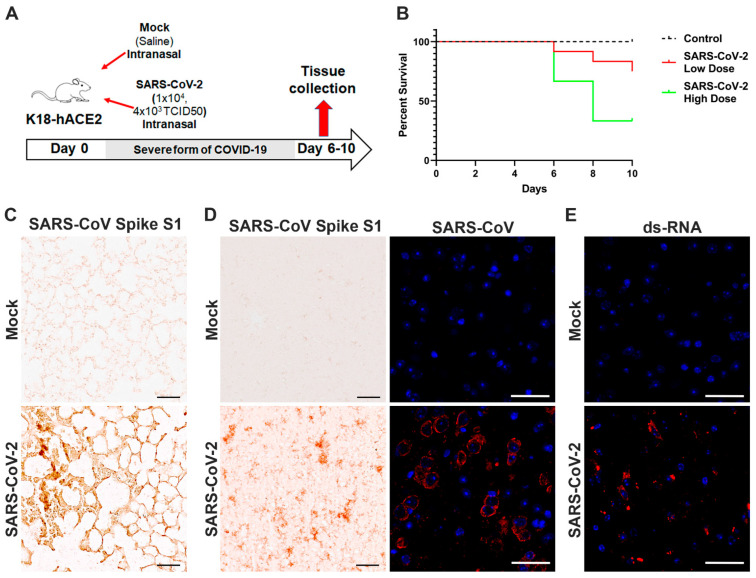
K18-hACE2 mice are susceptible to severe SARS-CoV-2 infection. (**A**) K18-hACE2 mice were intranasally infected with saline (mock control), 1 × 10^4^ or 4 × 10^3^ TCID50 SARS-CoV-2. (**B**) Survival curves of mice infected with 1 × 10^4^ (high dose) or 4 × 10^3^ (low dose) TCID50 SARS-CoV-2. All mice in the high dose group died (67%) or were euthanized by 10 dpi and diagnosed as having severe SARS-CoV-2. (**C**) Immunohistochemical detection of SARS-CoV-2 spike S1 protein antibody in lung tissue following mock and severe SARS-CoV-2 infection. (**D**) Detection of SARS-CoV-2 in olfactory bulb using two different antibodies, anti-SARS-CoV-2 spike S1 protein and anti-SARS-Co-V antibody, as described in methods. (**E**). Detection of double-stranded RNA (dsRNA) in olfactory bulb following severe SARS-CoV-2-infected mice compared to mock-infected mice, indicating active viral replication. Representative images from n = 3–6 mice/group. Scale bar, 100 μm.

To characterize the effects of SARS-CoV-2 infection on K18-hACE2 mice, we intranasally infected different groups of mice with either a low (4 × 10^3^ TCID50) or high (1 × 10^4^ TCID50) dose of SARS-CoV-2 virus (saline was used for mock-infected controls) (Figure 1A). Animals were monitored daily for clinical symptoms and body-weight changes. Mice that had rapid weight loss, lethargy, and became moribund between 6 and 10 dpi were euthanized. Sixty-seven percent of mice receiving a high dose, but only 20% of mice receiving a low dose, either died or were euthanized by 10 dpi. All mice in the high-dose group were euthanized by 10 dpi and diagnosed as having a severe form of SARS-CoV-2 infection (Figure 1B). Lung (Figure 1C) and brain olfactory bulb (Figure 1D) tissue from mice with a severe SARS-CoV-2 infection were immunostained with an antibody specific to the SARS-CoV-2 virus S protein. Robust expression of S protein was detected in both tissues at 10 dpi, but not in tissue from mock controls. In addition, detection of double-stranded RNA (dsRNA) in the olfactory bulb of SARS-CoV-2-infected mice compared to mock-infected mice, indicates active viral replication (Figure 1E). These data demonstrate that mice expressing hACE2 are highly susceptible to intranasal infection of SARS-CoV-2 virus and can develop a severe form of the disease.

### 3.2. Severe SARS-CoV-2 Infection Increases Inflammatory Markers in the Brain

**Figure 2:**
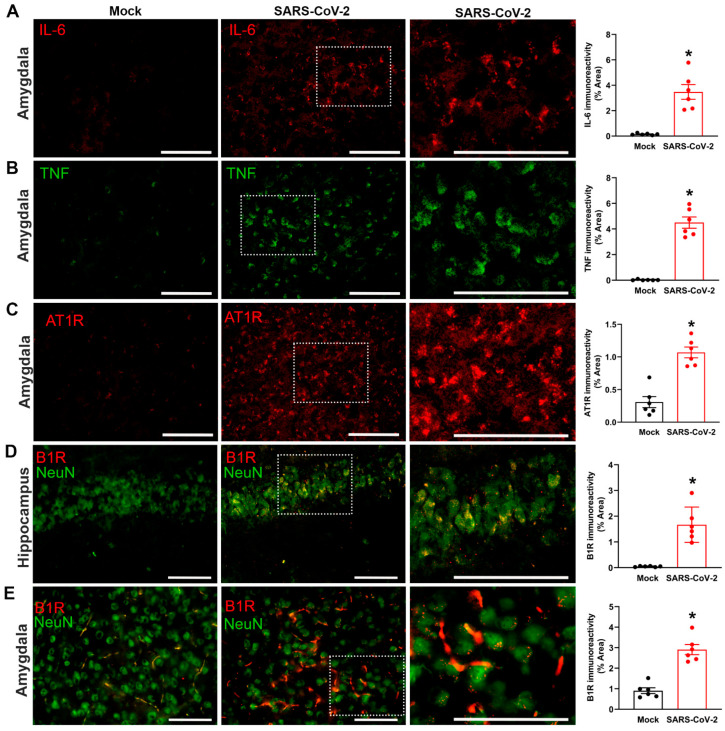
Severe SARS-CoV-2 infection increases inflammatory markers in the brain. (**A**–**C**) Immunofluorescence staining and quantification of IL-6, TNF, and AT1R immunoreactivity in the amygdala region of mock- and SARS-CoV-2-infected mice. The regions marked with white frame are magnified to the side. (**D**,**E**) Immunofluorescence staining and quantification of B1R immunoreactivity in the hippocampus and amygdala regions of mock- and SARS-CoV-2-infected mice. B1R expression (red) colocalizes with neurons stained with the cell specific marker NeuN (green). The regions marked with white frame are magnified to the side. Representative images from n = 6 mice/group. Scale bar, 50 μm. Data are mean ± s.e.m. * *p* < 0.05 (Student’s *t*-test).

We sought to determine whether severe SARS-CoV-2 infection produces inflammatory changes in the brains of K18-hCAE2 mice. Expression of the pro-inflammatory cytokines IL-6 and TNF, as well as the angiotensin II type 1 receptor (AT1R; the vasoactive pro-inflammatory component of the renin–angiotensin system), were measured in the amygdala region of the brain. Quantification of immunofluorescence staining revealed that, compared to mock controls, SARS-CoV-2-infected K18-hCAE2 mice showed increased expression of IL-6, TNF, and AT1R in the amygdala (Figure 2A–C). Because ACE2 can inactivate DABK, the ligand for pro-inflammatory B1Rs, binding of SARS-CoV-2 to ACE2 can dysregulate the bradykinin system. We therefore measured B1R expression in the brains of K18-hCAE2 mice. Immunofluorescence staining revealed significantly higher B1R immunoreactivity in neurons within the hippocampus and amygdala following SARS-CoV-2 infection, compared to mock controls (Figure 2D,E). Together, these data reveal a greater state of inflammation in the brains of SARS-CoV-2-infected hACE2 mice, corroborating data from COVID-19 patients with severe disease.

### 3.3. Mild SARS-CoV-2 Infection Causes Significant Antibody Generation 

**Figure 3:**
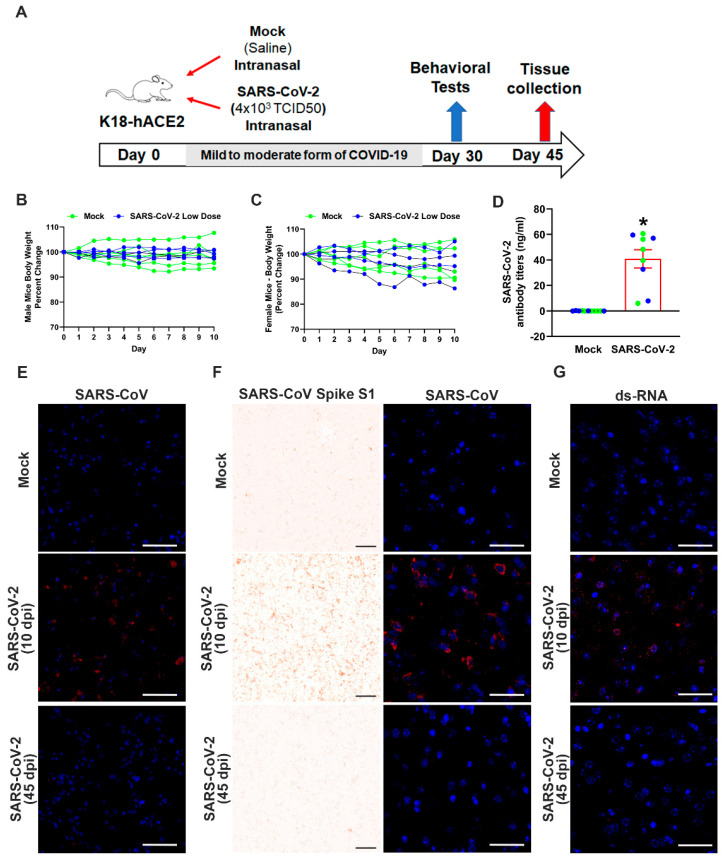
Mild SARS-CoV-2 infection causes significant antibody generation. (**A**) K18-hACE2 mice were intranasally infected with saline (mock control) or 4 × 10^3^ TCID50 SARS-CoV-2 and monitored for 45 days. Behavioral tests were performed from 30 dpi and mice were euthanized at 45 dpi. Eighty percent of mice survived this infection and were diagnosed as having mild disease, representing long COVID. (**B**,**C**) Infection had no effect on body weight in male (**B**) or female (**C**) mice. (**D**) High SARS-CoV-2 antibody titers detected in infected mice compared to mock controls at 45 dpi. n = 4 males and 5 females/group. Data are mean ± s.e.m. * *p* < 0.05 (Student’s *t*-test). Blue data points represent male mice, green data points represent female mice. Immunohistochemical staining of SARS-CoV-2 virus in lung tissue (**E**), with SARS-CoV serum) and olfactory bulb (**F**), with SARS-CoV-2 Spike S1 subunit antibody and SARS-CoV serum) of mock- or virus-infected mice, revealing staining for virus at 10 dpi and no detectable virus staining at 45 dpi. (**G**) Detection of double-stranded RNA (dsRNA) in olfactory bulb tissue in mild SARS-CoV-2-infected mice compared to mock-infected mice, indicating active viral replication at 10 dpi but not at 45 dpi. Scale bars, 50 μm.

To investigate the mechanisms responsible for the chronic neurological effects of SARS-CoV-2, we sought to create a robust model of long COVID. We intranasally infected K18-hACE2 mice with 4 × 10^3^ TCID50 of SARS-CoV-2 virus – a dose that gave rise to 80% survival (Figure 1B). This dose caused a mild SARS-CoV-2 infection, which we monitored on a daily basis for 45 days (Figure 3A). Clinical symptoms were assessed throughout this period and body weight changes were monitored for the first 10 days. We had implanted temperature microchips into the mice to determine whether SARS-CoV-2 infection produced a febrile response similar to humans, so that fever could be used as a clinical indicator of infection. However, the mice did not develop a fever with infection. We confirmed that 80% of infected mice survived the infection, showed no differences in body weight during the experimental period (45 dpi), and were diagnosed as having a mild SARS-CoV-2 infection (Figure 3B,C). Plasma samples from blood taken at 45 dpi showed that infected mice had high levels of IgG antibodies to SARS-CoV-2, whereas no antibodies were detected in mock controls (Figure 3D). In addition, immunostaining of lung and olfactory bulb tissue harvested at 10 dpi showed the presence of viral S protein expression, indicating the presence of virus in these regions (Figure 3E,F). However, immunostaining of lung and olfactory bulb tissue harvested at 45 dpi revealed the absence of viral S protein expression in either mock- or SARS-CoV-2-infected mice (Figure 3E,F). Detection of double-stranded RNA (dsRNA) in olfactory bulb tissue in mild SARS-CoV-2-infected mice compared to mock-infected mice indicated active viral replication at 10 dpi but not at 45 dpi (Figure 3G). Furthermore, the hippocampus, amygdala, and prefrontal cortex also showed the presence of virus or dsRNA at 10 dpi (Figure S1). Thus, recovery from a mild SARS-CoV-2 infection results in significant antibody generation that is not dependent on persistent viral replication.

### 3.4. Mild SARS-CoV-2 Infection Causes Lung Fibrosis and Increased B1R Expression

**Figure 4:**
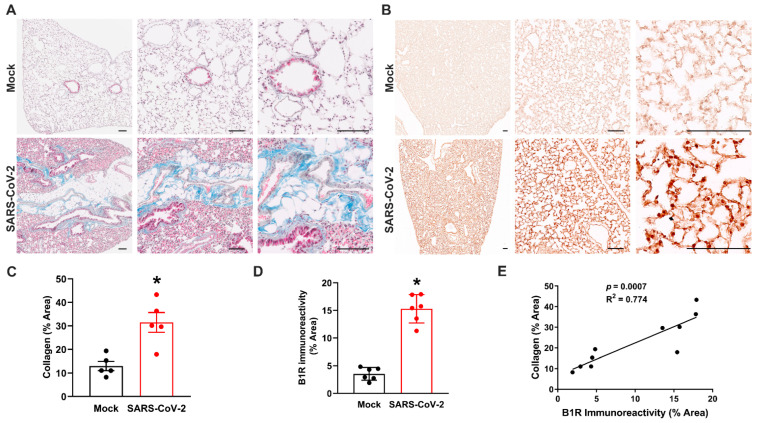
Mild SARS-CoV-2 infection causes lung fibrosis and increased B1R expression. (**A**) Masson’s trichrome staining of paraffin-embedded lung sections from K18-hACE2 mice at 45 dpi reveal increased collagen deposition (blue) and immune cell infiltration (red) in infected but not mock-treated animals. (**B**) Immunohistochemical staining of B1R (brown) in lung tissue of mice recovered from mild SARS-CoV-2 infection. Representative images from n = 5–6 mice per group. Scale bars, 100 μm. (**C**,**D**) Quantification data showing collagen (**C**) and B1R (**D**) expression in lungs. Representative images n = 9–10/group. Data are mean ± s.e.m. * *p* < 0.05 (Student’s *t*-test). (**E**) Pearson correlation and least-squares linear regression were used to determine correlation coefficients between variables. B1R immunoreactivity is positively correlated with collagen deposition in lungs infected with mild SARS-CoV-2 infection at 45 dpi.

One of the hallmarks of SARS-CoV-2 infection is lung fibrosis [33,34]. We therefore assessed the lungs of K18-hACE2 mice that had recovered from mild SARS-CoV-2 infection to validate the applicability of the animal model to long COVID. Collagen deposition was used as an indicator of lung fibrosis and was measured using Masson’s trichrome staining (Figure 4A). Quantification of this staining revealed greater collagen content in the lungs of SARS-CoV-2-infected mice at 45 dpi, compared to mock controls, indicating increased and long-lasting fibrosis (Figure 4C). Next, we measured the expression of pro-inflammatory B1Rs in lung sections using immunohistochemistry (Figure 4B). These data showed higher B1R expression following recovery from mild SARS-CoV-2 infection, compared to mock-infected mice (Figure 4D). To determine whether B1R expression levels in the lung are correlated with fibrosis, we performed linear regression analysis. This analysis revealed a statistically significant correlation between fibrosis and B1R expression in lung sections from SARS-CoV-2-infected mice (Figure 4E). Together, these data show that our mouse model of mild SARS-CoV-2 infection has long-lasting hallmarks of COVID-19.

### 3.5. Mild SARS-CoV-2 Infection Increases Inflammatory Markers in the Brain 

**Figure 5:**
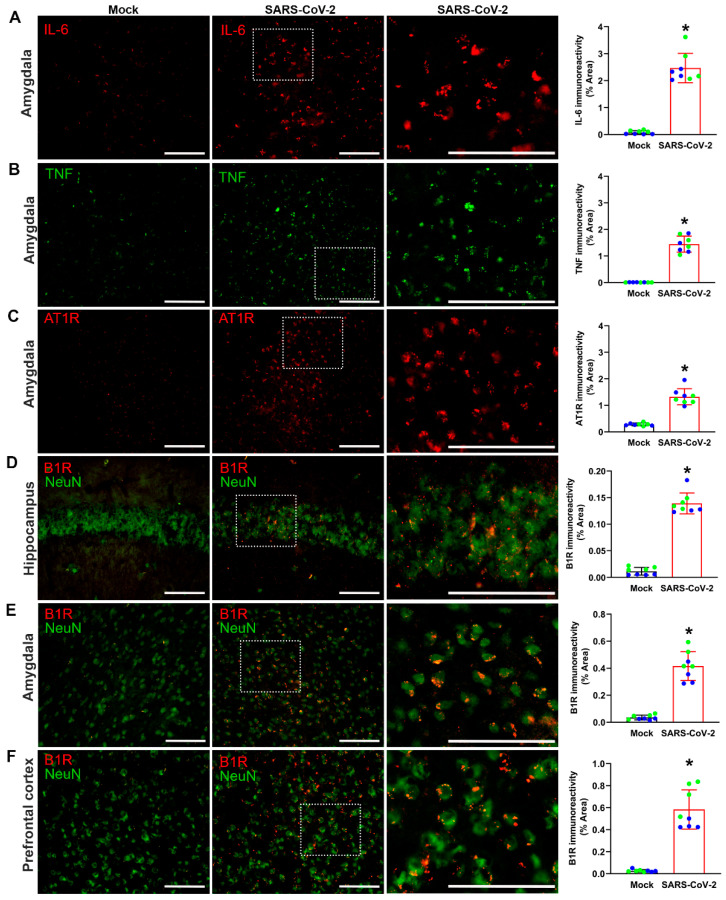
Mild SARS-CoV-2 infection increases inflammatory markers in the brain. (**A**–**C**) Immunofluorescence images and quantification of IL-6, TNF, and AT1R immunostaining in the amygdala of mock- and mild SARS-CoV-2-infected mice. The regions marked with white frame are magnified to the side. (**D**–**F**) Immunofluorescence staining and quantification of B1R immunoreactivity in the hippocampus, amygdala, and prefrontal cortex regions of mock- and SARS-CoV-2-infected mice at 45 dpi. The regions marked with white frame are magnified to the side. B1R expression (red) colocalizes with neurons stained with the cell specific marker NeuN (green). Representative images from n = 4 male and 4 female mice/group. Blue data points represent male mice, green data points represent female mice. Data are mean ± s.e.m. * *p* < 0.05 (Student’s *t*-test). Scale bar, 50 μM.

We next examined inflammatory changes in the brains of K18-hACE2 mice with mild SARS-CoV-2 infection (long COVID mice) to determine whether neuroinflammation is associated with the long-term effects of SARS-CoV-2 infection. Immunofluorescence staining revealed increased expression of IL-6 (Figure 5A), TNF (Figure 5B), and AT1R (Figure 5C) in the amygdala of these mice, compared to mock-infected controls. We also measured the expression of pro-inflammatory B1R expression in the brain regions that are associated with cognitive and neuropsychiatric symptoms. Quantification of immunofluorescence at 45 dpi revealed that B1R expression was significantly increased in the hippocampus (Figure 5D), amygdala (Figure 5E), and prefrontal cortex (Figure 5F) of long COVID mice, compared to mock controls. These effects were qualitatively similar to those observed following severe SARS-CoV-2 infection (Figure 2), revealing that even mild SARS-CoV-2 infection can lead to long-term inflammation in the CNS. To further validate the elevated expression of inflammatory markers demonstrated with immunofluorescence, we used real-time qRT-PCR to measure mRNA levels. Significant elevations in IL-6, TNF, AT1R, and B1R were found in hippocampus and prefrontal cortex 45 dpi (Figure S2), which supported our findings that increased neuroinflammation is associated with the long-term effects of SARS-CoV-2 infection. The expression of B1R seems to be co-localized with neurons (Figure 5D–F) and with microglia (Figure S3).

### 3.6. Mild SARS-CoV-2 Infection Results in Persistent Activation of Microglia in the Brain 

**Figure 6:**
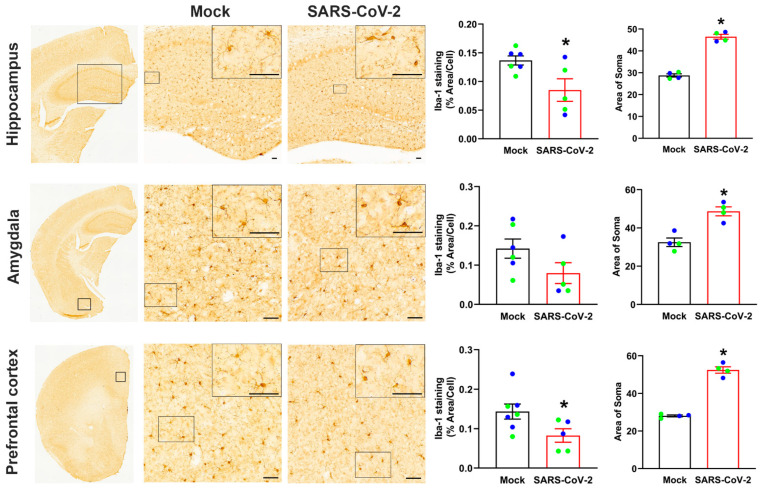
Mild SARS-CoV-2 infection increases microglia activation in the brain. Immunohistochemistry images showing the staining for Iba-1 in hippocampus, amygdala, and prefrontal cortex of mock- and SARS-CoV-2-infected mice. A high magnification image of the boxed region is shown in the top right of each image. The microglia in the hippocampus and prefrontal cortex in mice recovered from SARS-CoV-2 infection had a signification reduction in microglia processes, based on area of Iba-1 staining relative to the number of microglia cell bodies, indicating microglial activation. A significant increase in the average size of the microglia cell bodies, measured as area of soma, was observed in all regions investigated. Representative images from n = 2–4 male and 2–4 female mice/group. Blue data points represent male mice, green data points represent female mice. Data are mean ± s.e.m. * *p* < 0.05 (Student’s *t*-test). Scale bar, 50 μM.

Microglia have a complex and diverse morphologic response to inflammation and in acute conditions can proliferate and infiltrate the affected area, resulting in an increase in the number of microglia. However, more subtle changes in microglia morphology are associated with chronic inflammation [35]. Microglia morphology can range from resting microglia with complex, hyper-ramified processes, to activated de-ramified microglia and amoeboid-shaped morphologies. Because these changes in morphology can serve as an indicator of microglia function and signal long-term dysfunction in the brain, we analyzed Iba1 immunohistochemistry to assess microglia activation in the hippocampus, amygdala, and prefrontal cortex following recovery from SARS-CoV-2 infection (long COVID mice). No differences in the number of microglia were observed. The microglia in the hippocampus and prefrontal cortex in mice that recovered from SARS-CoV-2 infection had a significant reduction in microglia processes, determined based on overall area of Iba1 staining as well as increased staining of microglia soma, indicating microglia activation (Figure 6). A similar trend was found in the amygdala; however, this was not statistically significant. This microglia activation was further confirmed by evaluating microglia morphology using skeletal analysis which revealed reduced microglia processes within the amygdala (Figure S4).

### 3.7. Mild SARS-CoV-2 Infection Causes Neuropsychiatric and Behavioral Symptoms 

**Figure 7:**
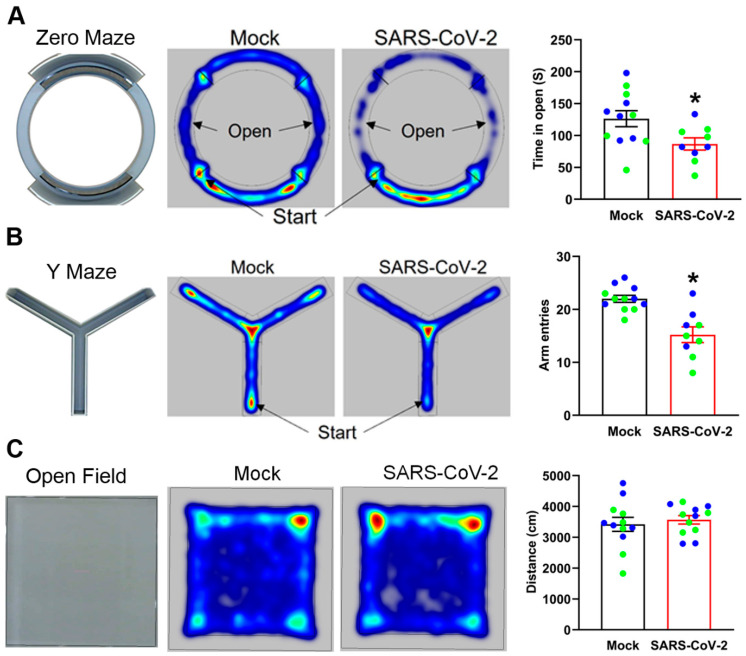
Mild SARS-CoV-2 infection causes neuropsychiatric and cognitive symptoms. (**A**) Mock- and SARS-CoV-2-infected K18-hACE2 mice were tested from 30 dpi in the zero maze. Representative heat maps show that infected mice spent significantly less time in the open portions of the maze, defined as any part of the mouse entering the open portion of the maze. (**B**) Mock- and SARS-CoV-2-infected K18-hACE2 mice were tested from 30 dpi in the Y maze. Heat maps show that infected mice displayed significantly reduced arm exploration. (**C**) Mock- and SARS-CoV-2-infected K18-hACE2 mice were tested from 30 dpi in the open field test. Heat maps show total distance traveled by mice over the whole area of the apparatus, indicating no differences in overall motor activity. Blue data points represent male mice, green data points represent female mice. n = 6 male and 6 female mice in each group. Data are mean ± s.e.m. * *p* < 0.05 (Student’s *t*-test).

To investigate the long-term effects of mild SARS-CoV-2 infection on mouse behavior, we tested our long COVID mice in the zero maze, the Y maze, and an open field, between 30 and 45 dpi. We used the zero maze to assess anxiety. Both partial and full-body entries into the open, as well as the duration of full-body entries, were reduced, but not significantly different, in infected mice compared to mock controls (Table 1). However, long COVID mice spent significantly less time in the open areas of the zero maze than mock-infected mice, indicating elevated levels of anxiety (Figure 7A). We also tested the behavior of mice in a Y maze to assess working spatial memory and exploratory behavior. Mock- and SARS-CoV-2-infected mice performed similarly on % spontaneous alternations of the Y maze, indicating comparable working memory. Infected mice spent more time in the center of the maze, and were more immobile, but these parameters were not statistically significant (Table 1). However, long COVID mice showed a significant reduction in the total number of arm entries (Figure 7B), as well as average velocity and distance traveled (Table 1), indicating diminished exploratory behavior. The open field test was used to determine overall exploratory and motor activity. No differences were observed in distance traveled, acceleration, or immobility between long COVID and mock-infected mice (Table 1). In addition, there were no differences in the time spent near walls of the open field, a crude indicator of anxiety. Representative heat maps from a subset of mice show the total distance traveled by mice over the whole area of the apparatus (Figure 7C). The open field results established that infection does not alter overall motor activity in our mouse model of long COVID. Together, these data suggest that mild SARS-CoV-2 infection causes persistent neurological symptoms, including elevated anxiety and reduced exploratory behavior. 

## 4. Discussion

COVID-19 has a broad spectrum of symptoms in humans, ranging from asymptomatic to severe disease. Long COVID or PASC is being diagnosed not only in patients having recovered from severe COVID-19 but also in patients with mild symptoms and asymptomatic cases. Because patients are reporting long-lasting symptoms, including cognitive, neurological, and neuropsychiatric following infection with SARS-CoV-2, it is important to understand the causative mechanisms. Potential mechanisms have not yet been investigated in animal models of COVID-19. Although previous studies have investigated the acute response of K18-hACE2 mice to infection with SARS-CoV-2, our study is the first to characterize longer-term effects on behavior and brain inflammation following recovery from mild disease. Our results indicate that: (1) both acute severe disease and chronic mild disease can result from SARS-CoV-2 infection of K18-hACE2 mice; (2) SARS-CoV-2 infection increases the expression of pro-inflammatory mediators IL-6, TNF, AT1R, and B1R in the brain following mild disease (45 dpi) as well as during severe disease (10 dpi); (3) mild SARS-CoV-2 infection induces fibrosis and elevates B1R expression in the lungs of K18-hACE2 mice; (4) behavioral changes are associated with recovery from mild SARS-CoV-2 infection in mice, mimicking some long COVID symptoms in humans; and (5) elevated B1R expression could be a critical mechanism driving inflammation in the brain following SARS-CoV-2 infection.

K18-hACE2 mice have been primarily used to study the acute phase of COVID-19. Our study further reveals that the K18-hACE2 mouse model represents an important model to study the physiological effects of SARS-CoV-2. The advantages of this model are that it reproduces a wide diversity of symptoms, ranging from mild/asymptomatic cases to lethal infection that are not replicated in most mouse models of COVID-19. At high doses of SARS-CoV-2, 100% mortality has been reported in K18-hACE2 mice [36,37]. However, mortality has been reported to decrease at lower doses [36]. In order to study the long-term effects of SARS-CoV-2 infection, we investigated a dose that produces a mild infection and only 20% mortality. Nevertheless, the mice had antibodies to SARS-CoV-2 and lung fibrosis at 45 dpi, demonstrating an infection that mimicked long COVID in humans. Furthermore, this model replicated transient viral infection in the brain, which is absent in some mouse models but demonstrated in many human cases [15,16,18,38]. Thus, this model replicates high susceptibility to the effects of SARS-CoV-2 infection and is a valuable tool to evaluate the long-term effects of SARS-CoV-2 infection on brain inflammation and behavioral changes.

Recent findings suggest that alterations of kinins in the kallikrein–kinin system (KKS), known as a bradykinin storm, play a more prominent role in multiple organ system damage leading to severe COVID-19 symptoms [3,4,19]. The KKS is composed of polypeptide kallikreins that can proteolytically cleave high- and low-molecular-weight kininogens to release the vasoactive peptides bradykinin and kallidin. In addition, the peptidase kininase I further processes bradykinin and kallidin into the active metabolites DABK and des-Arg^10^-kallidin (DAKD), respectively. These various kinins transmit their biological effects by activating the G-protein coupled receptors bradykinin/kinin receptor 1 (B1R) and 2 (B2R). Bradykinin and kallidin bind to ubiquitously expressing B2R, whereas DABK and DAKD bind to inducible B1R. In our study, we found elevated expression of pro-inflammatory mediators (B1R and AT1R) in the brain following severe infection with SARS-CoV-2, reproducing the disrupted KKS signaling reported in COVID-19 patients. Considerably fewer data are available on the mechanisms associated with persistent SARS-CoV-2 symptoms following mild infection that result in long COVID. Interestingly, these pro-inflammatory mediators were also elevated in the brain up to 45 days following mild infection. These data suggest that disruption of the KKS signaling and induction of B1R could occur in patients following mild SARS-CoV-2 infection and with long COVID, as well as following severe COVID.

Currently, K18-hACE2 mice replicate the capability of SARS-CoV-2 to infect the brain, as previous studies have found evidence of SARS-CoV-2 in the brains of these mice after infection with the virus [18,39]. These data were primarily based on infections that resulted in severe disease. Our data show that SARS-CoV-2 does enter the brain in K18-hACE2 mice with mild disease based on the presence of the virus at 10 dpi, but does not persist at 45 dpi. Additionally, data from these mice show clear and persistent effects on brain inflammation and behavior that last up to 45 days. This transient infection in the brain could contribute to the persistent inflammation. However, an indirect effect on the brain is also possible. In hamsters, Klein et al. reported elevated cytokine expression following SARS-CoV-2 infection in the absence of evidence for virus in the brain [40]. However, the elevated cytokine expression (IL-1β and IL-6) and microglial activation in this model was transient, and values returned to control levels between 5 and 8 dpi. In contrast, Fernández-Castañeda et al., using intranasal adeno-associated virus expression of hACE2 which lacks neuro-invasion, found persistent elevation of inflammatory markers in the brain and reduced neurogenesis in the hippocampus up to 7 weeks post infection [38]. More recently, elevated microglia activation, neuroinflammation, and evidence of neurodegeneration have been reported 4–6 weeks after SARS-CoV-2 infection in non-human primates [41,42]. Peripheral immune activation or autoantibodies could contribute to effects observed in the brain in the absence of direct infiltration of the CNS. However, none of these mechanisms are mutually exclusive. Increased pro-inflammatory cytokines could disrupt the blood brain barrier (BBB), allowing the entry of virus, cytokines, and/or autoantibodies to the CNS. In humans with long COVID, the variability in persistent symptoms could be due to differences in the activation of these mechanisms. For example, direct infiltration into the brain could result in long-lasting inflammation and neurological symptoms, whereas an inability of the virus to access the brain may explain why some patients do not exhibit persistent symptoms [43,44]. Currently, the mechanisms involved in SARS-CoV-2 infection of the CNS have not been determined fully. 

In our study, we found upregulated pro-inflammatory kinin B1R expression in the amygdala, hippocampus, and prefrontal cortex, both in severe as well as mild, long-term disease. Given the role of B1R in increasing the inflammatory state, we speculate that increased activation of B1R could lead to elevated inflammation in the brain and contribute to the observed behavioral changes. B1R has the potential to mediate the chronic actions of the KKS system. Once B1R is activated by an agonist, it is not internalized or desensitized, and perpetuates the feed-forward cycle of neuroinflammation. Additionally, all components of the KKS can be synthesized locally in the cerebral cortex, brain stem, cerebellum, hypothalamus, and hippocampus, among other areas, implying that induction of B1R expression could result from either a peripheral mechanism or central activation [45]. Indeed, significant increases in B1R expression have been reported in various traumatic brain injury models, the longest lasting at least 7 d from contusion [46,47,48]. Additionally, traumatic brain injury and repeated dosing models of infection such as lipopolysaccharide or poly(I:C) can cause elevation of inflammatory markers for up to 30 days [49,50,51]. Therefore, the persistent effects of mild COVID-19 on B1R expression and inflammation in K18-hACE2 mice appears to be similar or longer lasting than traumatic brain injury and other high-dose inflammation models.

Our study investigated the behavior of mice that had recovered from a mild SARS-CoV-2 infection. Based on the open field test, there were no overt effects on exploration, motor function, or anxiety. However, SARS-CoV-2 infection enhanced anxiety-related behavior in the zero maze and attenuated exploratory behavior in the Y maze. It is unclear whether altered behavior in the Y maze is due to a reduced drive for exploration, elevated anxiety, or potential attention deficits. No differences in % spontaneous alternations were observed between groups. However, this parameter is difficult to interpret due significantly fewer arm entries in the infected mice. Since open field activity was not affected and the virus was cleared from the brain and lungs, these data suggest that the persistent inflammation resulting from infection is a cause of these behavioral changes. Induction of brain inflammation using models that mimic infection, such as lipopolysaccharide and poly(I:C), produce cognitive deficits, enhance anxiety, and exacerbate depression-related behaviors. More specifically, studies involving repeated exposure to silicon dioxide nanoparticles (which produce inflammation), endotoxin-induced nigrostriatal damage, and maternal immune activation using poly(I:C), reported attenuation of Y maze exploration [52,53,54,55]. Maternal immune activation also elevated anxiety measures [54]. Additional research is needed to differentiate between the general effects of infection-induced immune activation and the specific mechanism associated with SARS-CoV-2 infection.

In our long COVID model utilizing K18-hACE2 mice, we observed lung damage, elevated neuroinflammation, and neuropsychiatric symptoms. Lung damage and deficits in lung function have been reported in long COVID patients [56,57]. Furthermore, MRI and PET studies have reported persistent deficits and enhanced markers of inflammation in the brains of patients recovered from COVID-19 [58,59]. Common cognitive and neuropsychiatric symptoms in long COVID patients include fatigue, depression, anxiety, and memory problems [7,8,60]. However, the cause of these persistent symptoms is currently unclear. Our data reveal that K18-hACE2 mice following mild SARS-CoV-2 infection is a useful model to study the long-term effects of COVID. Because the effects of SARS-CoV-2 infection in humans are variable, from asymptomatic to lethal, this model likely reproduces aspects of the disease that occur in a subset of patients most sensitive to the effects of SARS-CoV-2 infection. Further validation is needed to determine if the KKS and B1R expression is altered in the brains of patients with long COVID.

An additional consideration associated with persistent inflammation is the increased likelihood of developing disease in the future. Chronically elevated inflammation has been associated with neurodegenerative diseases such as Parkinson’s, Alzheimer’s, and other dementias [10,11,12,61,62,63]. Although individuals with long COVID likely represent those with CNS inflammation and who are therefore at greater risk of future neurodegenerative disease, others with prior SARS-CoV-2 infections could also have an elevated risk. The complications associated with clinical data, including diverse genetics, confounding comorbidities, different pathogen exposures, and immune memory, create serious challenges for elucidating biological mechanisms and developing potential treatments. These confounding variables can be controlled in animal models to gain an understanding of the most relevant mechanisms and provide a means to test promising therapies. 

## 5. Conclusions

In conclusion, our study has clearly demonstrated persistent elevation of brain inflammation in a mouse model of long COVID and elevated expression of B1R. Due to the role of B1R in mediating inflammation, these results suggest that B1R could be a useful therapeutic target to attenuate COVID-19-associated inflammation. In future studies, the role of B1R signaling can be tested to determine the contribution to the persistent inflammation in this mouse model of long COVID. A further understanding of B1R-mediated signaling could lead to the identification of additional therapeutic targets, which may have implications for the prevention of neurodegeneration following long COVID.

## Figures and Tables

**Table 1:** Behavioral parameters in the open field, zero maze, and Y maze 30 days after SARS-CoV-2 infection.

	Mock	SARS-CoV-2
Open field		
Distance (cm)	3422.17 ± 227.39	3443.96 ± 178.66
Velocity (cm/s)	5.70 ± 0.38	5.54 ± 0.36
Acceleration (cm/s^2^)	669.95 ± 29.46	664.50 ± 35.79
Time moving (s)	348.27 ± 18.84	364.01± 16.43
Time in border (s)	549.43 ± 16.65	529.06 ± 14.17
		
**Zero Maze**		
Entries	19.25 ± 1.87	16.33 ± 1.69
Entries, whole body	4.58 ± 1.09	2.33 ± 0.92
Time in open, whole body (s)	35.56 ± 7.26	18.24 ± 7.31
		
**Y Maze**		
Spontaneous alternation (%)	65.13 ± 2.85	64.95 ± 4.92
Immobile (s)	9.37 ± 2.76	24.80 ± 10.34
Velocity (cm/s)	4.24 ± 0.15	3.05 ± 0.27 *
Distance (cm)	1272.66 ± 43.51	916.3 ± 79.37 *

* *p* < 0.05 (Student’s *t*-test).

## Data Availability

All data supporting the findings of this study are available within the paper and its Supplementary Information.

## Supplementary Materials

The following are available online at https://www.mdpi.com/article/10.3390/cells12162107/s1, Table S1: Primer sequences used for real-time quantitative reverse transcriptase polymerase chain reaction (qRT-PCR), Figure S1: SARS-CoV-2 infected K18-hACE2 mice show presence of virus in the brain, Figure S2: SARS-CoV-2 infection increases inflammatory markers in the brain, Figure S3: Co-localized expression of B1R with microglia, Figure S4: Mild infection with SARS-CoV-2 and microglia ramifications in different brain regions.

## References

[1] Larsen J.R., Martin M.R., Martin J.D., Kuhn P., Hicks J.B. (2020). Modeling the Onset of Symptoms of COVID-19. Front. Public Health.

[2] Buszko M., Park J.H., Verthelyi D., Sen R., Young H.A., Rosenberg A.S. (2020). The dynamic changes in cytokine responses in COVID-19: A snapshot of the current state of knowledge. Nat. Immunol..

[3] Roche J.A., Roche R. (2020). A hypothesized role for dysregulated bradykinin signaling in COVID-19 respiratory complications. FASEB J..

[4] Garvin M.R., Alvarez C., Miller J.I., Prates E.T., Walker A.M., Amos B.K., Mast A.E., Justice A., Aronow B., Jacobson D. (2020). A mechanistic model and therapeutic interventions for COVID-19 involving a RAS-mediated bradykinin storm. eLife.

[5] Logue J.K., Franko N.M., McCulloch D.J., McDonald D., Magedson A., Wolf C.R., Chu H.Y. (2021). Sequelae in Adults at 6 Months After COVID-19 Infection. JAMA Netw. Open.

[6] Proal A.D., VanElzakker M.B. (2021). Long COVID or Post-acute Sequelae of COVID-19 (PASC): An Overview of Biological Factors That May Contribute to Persistent Symptoms. Front. Microbiol..

[7] Taquet M., Geddes J.R., Husain M., Luciano S., Harrison P.J. (2021). 6-month neurological and psychiatric outcomes in 236 379 survivors of COVID-19: A retrospective cohort study using electronic health records. Lancet Psychiatry.

[8] Graham E.L., Clark J.R., Orban Z.S., Lim P.H., Szymanski A.L., Taylor C., DiBiase R.M., Jia D.T., Balabanov R., Ho S.U. (2021). Persistent neurologic symptoms and cognitive dysfunction in non-hospitalized Covid-19 "long haulers". Ann. Clin. Transl. Neurol..

[9] Reiken S., Sittenfeld L., Dridi H., Liu Y., Liu X., Marks A.R. (2022). Alzheimer’s-like signaling in brains of COVID-19 patients. Alzheimers Dement..

[10] Cocoros N.M., Svensson E., Szepligeti S.K., Vestergaard S.V., Szentkuti P., Thomsen R.W., Borghammer P., Sorensen H.T., Henderson V.W. (2021). Long-term Risk of Parkinson Disease Following Influenza and Other Infections. JAMA Neurol..

[11] Vigasova D., Nemergut M., Liskova B., Damborsky J. (2021). Multi-pathogen infections and Alzheimer’s disease. Microb Cell Fact..

[12] Tarlinton R.E., Martynova E., Rizvanov A.A., Khaiboullina S., Verma S. (2020). Role of Viruses in the Pathogenesis of Multiple Sclerosis. Viruses.

[13] Guedj E., Million M., Dudouet P., Tissot-Dupont H., Bregeon F., Cammilleri S., Raoult D. (2021). (18)F-FDG brain PET hypometabolism in post-SARS-CoV-2 infection: Substrate for persistent/delayed disorders?. Eur. J. Nucl. Med. Mol. Imaging.

[14] Yong S.J. (2021). Long COVID or post-COVID-19 syndrome: Putative pathophysiology, risk factors, and treatments. Infect Dis..

[15] Liu J.M., Tan B.H., Wu S., Gui Y., Suo J.L., Li Y.C. (2021). Evidence of central nervous system infection and neuroinvasive routes, as well as neurological involvement, in the lethality of SARS-CoV-2 infection. J. Med. Virol..

[16] Lewis A., Frontera J., Placantonakis D.G., Lighter J., Galetta S., Balcer L., Melmed K.R. (2021). Cerebrospinal fluid in COVID-19: A systematic review of the literature. J. Neurol. Sci..

[17] Stein S.R., Ramelli S.C., Grazioli A., Chung J.Y., Singh M., Yinda C.K., Winkler C.W., Sun J., Dickey J.M., Ylaya K. (2022). SARS-CoV-2 infection and persistence in the human body and brain at autopsy. Nature.

[18] Song E., Zhang C., Israelow B., Lu-Culligan A., Prado A.V., Skriabine S., Lu P., Weizman O.E., Liu F., Dai Y. (2021). Neuroinvasion of SARS-CoV-2 in human and mouse brain. J. Exp. Med..

[19] Savitt A.G., Manimala S., White T., Fandaros M., Yin W., Duan H., Xu X., Geisbrecht B.V., Rubenstein D.A., Kaplan A.P. (2021). SARS-CoV-2 Exacerbates COVID-19 Pathology Through Activation of the Complement and Kinin Systems. Front. Immunol..

[20] Jia H., Yue X., Lazartigues E. (2020). ACE2 mouse models: A toolbox for cardiovascular and pulmonary research. Nat. Commun..

[21] Dinnon K.H., Leist S.R., Schafer A., Edwards C.E., Martinez D.R., Montgomery S.A., West A., Yount B.L., Hou Y.J., Adams L.E. (2020). A mouse-adapted model of SARS-CoV-2 to test COVID-19 countermeasures. Nature.

[22] Bao L., Deng W., Huang B., Gao H., Liu J., Ren L., Wei Q., Yu P., Xu Y., Qi F. (2020). The pathogenicity of SARS-CoV-2 in hACE2 transgenic mice. Nature.

[23] Yinda C.K., Port J.R., Bushmaker T., Offei Owusu I., Purushotham J.N., Avanzato V.A., Fischer R.J., Schulz J.E., Holbrook M.G., Hebner M.J. (2021). K18-hACE2 mice develop respiratory disease resembling severe COVID-19. PLoS Pathog..

[24] Sun S.H., Chen Q., Gu H.J., Yang G., Wang Y.X., Huang X.Y., Liu S.S., Zhang N.N., Li X.F., Xiong R. (2020). A Mouse Model of SARS-CoV-2 Infection and Pathogenesis. Cell Host Microbe.

[25] Dong W., Mead H., Tian L., Park J.G., Garcia J.I., Jaramillo S., Barr T., Kollath D.S., Coyne V.K., Stone N.E. (2022). The K18-Human ACE2 Transgenic Mouse Model Recapitulates Non-severe and Severe COVID-19 in Response to an Infectious Dose of the SARS-CoV-2 Virus. J. Virol..

[26] Carossino M., Kenney D., O’Connell A.K., Montanaro P., Tseng A.E., Gertje H.P., Grosz K.A., Ericsson M., Huber B.R., Kurnick S.A. (2022). Fatal Neurodissemination and SARS-CoV-2 Tropism in K18-hACE2 Mice Is Only Partially Dependent on hACE2 Expression. Viruses.

[27] Parekh R.U., Robidoux J., Sriramula S. (2020). Kinin B1 Receptor Blockade Prevents Angiotensin II-induced Neuroinflammation and Oxidative Stress in Primary Hypothalamic Neurons. Cell Mol. Neurobiol..

[28] Akula S.M., Bolin P., Cook P.P. (2022). Cellular miR-150-5p may have a crucial role to play in the biology of SARS-CoV-2 infection by regulating nsp10 gene. RNA Biol..

[29] Young K., Morrison H. (2018). Quantifying Microglia Morphology from Photomicrographs of Immunohistochemistry Prepared Tissue Using ImageJ. J. Vis. Exp..

[30] Morrison H., Young K., Qureshi M., Rowe R.K., Lifshitz J. (2017). Quantitative microglia analyses reveal diverse morphologic responses in the rat cortex after diffuse brain injury. Sci. Rep..

[31] Parekh R.U., Sriramula S. (2020). Activation of Kinin B1R Upregulates ADAM17 and Results in ACE2 Shedding in Neurons. Int. J. Mol. Sci..

[32] Sriramula S., Lazartigues E. (2017). Kinin B1 Receptor Promotes Neurogenic Hypertension Through Activation of Centrally Mediated Mechanisms. Hypertension.

[33] Bazdyrev E., Rusina P., Panova M., Novikov F., Grishagin I., Nebolsin V. (2021). Lung Fibrosis after COVID-19: Treatment Prospects. Pharmaceuticals.

[34] John A.E., Joseph C., Jenkins G., Tatler A.L. (2021). COVID-19 and pulmonary fibrosis: A potential role for lung epithelial cells and fibroblasts. Immunol. Rev..

[35] Leon-Rodriguez A., Fernandez-Arjona M.D.M., Grondona J.M., Pedraza C., Lopez-Avalos M.D. (2022). Anxiety-like behavior and microglial activation in the amygdala after acute neuroinflammation induced by microbial neuraminidase. Sci. Rep..

[36] Golden J.W., Cline C.R., Zeng X., Garrison A.R., Carey B.D., Mucker E.M., White L.E., Shamblin J.D., Brocato R.L., Liu J. (2020). Human angiotensin-converting enzyme 2 transgenic mice infected with SARS-CoV-2 develop severe and fatal respiratory disease. JCI Insight.

[37] Oladunni F.S., Park J.G., Pino P.A., Gonzalez O., Akhter A., Allue-Guardia A., Olmo-Fontanez A., Gautam S., Garcia-Vilanova A., Ye C. (2020). Lethality of SARS-CoV-2 infection in K18 human angiotensin-converting enzyme 2 transgenic mice. Nat. Commun..

[38] Fernandez-Castaneda A., Lu P., Geraghty A.C., Song E., Lee M.H., Wood J., Yalcin B., Taylor K.R., Dutton S., Acosta-Alvarez L. Mild respiratory SARS-CoV-2 infection can cause multi-lineage cellular dysregulation and myelin loss in the brain. bioRxiv.

[39] Kumari P., Rothan H.A., Natekar J.P., Stone S., Pathak H., Strate P.G., Arora K., Brinton M.A., Kumar M. (2021). Neuroinvasion and Encephalitis Following Intranasal Inoculation of SARS-CoV-2 in K18-hACE2 Mice. Viruses.

[40] Klein R., Soung A., Sissoko C., Nordvig A., Canoll P., Mariani M., Jiang X., Bricker T., Goldman J., Rosoklija G. COVID-19 induces neuroinflammation and loss of hippocampal neurogenesis. Res. Sq..

[41] Philippens I., Boszormenyi K.P., Wubben J.A.M., Fagrouch Z.C., van Driel N., Mayenburg A.Q., Lozovagia D., Roos E., Schurink B., Bugiani M. (2022). Brain Inflammation and Intracellular alpha-Synuclein Aggregates in Macaques after SARS-CoV-2 Infection. Viruses.

[42] Rutkai I., Mayer M.G., Hellmers L.M., Ning B., Huang Z., Monjure C.J., Coyne C., Silvestri R., Golden N., Hensley K. (2022). Neuropathology and virus in brain of SARS-CoV-2 infected non-human primates. Nat. Commun..

[43] Chen Y., Yang W., Chen F., Cui L. (2022). COVID-19 and cognitive impairment: Neuroinvasive and blood—brain barrier dysfunction. J. Neuroinflamm..

[44] Krasemann S., Haferkamp U., Pfefferle S., Woo M.S., Heinrich F., Schweizer M., Appelt-Menzel A., Cubukova A., Barenberg J., Leu J. (2022). The blood-brain barrier is dysregulated in COVID-19 and serves as a CNS entry route for SARS-CoV-2. Stem Cell Rep..

[45] Chao J., Woodley C., Chao L., Margolius H.S. (1983). Identification of tissue kallikrein in brain and in the cell-free translation product encoded by brain mRNA. J. Biol. Chem..

[46] Albert-Weissenberger C., Stetter C., Meuth S.G., Gobel K., Bader M., Siren A.L., Kleinschnitz C. (2012). Blocking of bradykinin receptor B1 protects from focal closed head injury in mice by reducing axonal damage and astroglia activation. J. Cereb. Blood Flow Metab..

[47] Raslan F., Schwarz T., Meuth S.G., Austinat M., Bader M., Renne T., Roosen K., Stoll G., Siren A.L., Kleinschnitz C. (2010). Inhibition of bradykinin receptor B1 protects mice from focal brain injury by reducing blood-brain barrier leakage and inflammation. J. Cereb. Blood Flow Metab..

[48] Trabold R., Eros C., Zweckberger K., Relton J., Beck H., Nussberger J., Muller-Esterl W., Bader M., Whalley E., Plesnila N. (2010). The role of bradykinin B(1) and B(2) receptors for secondary brain damage after traumatic brain injury in mice. J. Cereb. Blood Flow Metab..

[49] Witcher K.G., Bray C.E., Chunchai T., Zhao F., O’Neil S.M., Gordillo A.J., Campbell W.A., McKim D.B., Liu X., Dziabis J.E. (2021). Traumatic Brain Injury Causes Chronic Cortical Inflammation and Neuronal Dysfunction Mediated by Microglia. J. Neurosci..

[50] Hoogland I.C., Houbolt C., van Westerloo D.J., van Gool W.A., van de Beek D. (2015). Systemic inflammation and microglial activation: Systematic review of animal experiments. J. Neuroinflamm..

[51] Cunningham C., Campion S., Teeling J., Felton L., Perry V.H. (2007). The sickness behaviour and CNS inflammatory mediator profile induced by systemic challenge of mice with synthetic double-stranded RNA (poly I:C). Brain Behav. Immun..

[52] Jarrar B., Al-Doaiss A., Shati A., Al-Kahtani M., Jarrar Q. (2021). Behavioural alterations induced by chronic exposure to 10 nm silicon dioxide nanoparticles. IET Nanobiotechnol..

[53] Li W., Chen M., Feng X., Song M., Shao M., Yang Y., Zhang L., Liu Q., Lv L., Su X. (2021). Maternal immune activation alters adult behavior, intestinal integrity, gut microbiota and the gut inflammation. Brain Behav..

[54] Kitanaka J., Kitanaka N., Hall F.S., Fujii M., Goto A., Kanda Y., Koizumi A., Kuroiwa H., Mibayashi S., Muranishi Y. (2015). Memory impairment and reduced exploratory behavior in mice after administration of systemic morphine. J. Exp. Neurosci..

[55] Darwish B., Chamaa F., Al-Chaer E.D., Saade N.E., Abou-Kheir W. (2019). Intranigral Injection of Endotoxin Suppresses Proliferation of Hippocampal Progenitor Cells. Front. Neurosci..

[56] Heiss R., Grodzki D.M., Horger W., Uder M., Nagel A.M., Bickelhaupt S. (2021). High-performance low field MRI enables visualization of persistent pulmonary damage after COVID-19. Magn. Reson. Imaging.

[57] Cho J.L., Villacreses R., Nagpal P., Guo J., Pezzulo A.A., Thurman A.L., Hamzeh N.Y., Blount R.J., Fortis S., Hoffman E.A. (2022). Quantitative Chest CT Assessment of Small Airways Disease in Post-Acute SARS-CoV-2 Infection. Radiology.

[58] Sollini M., Morbelli S., Ciccarelli M., Cecconi M., Aghemo A., Morelli P., Chiola S., Gelardi F., Chiti A. (2021). Long COVID hallmarks on [18F]FDG-PET/CT: A case-control study. Eur. J. Nucl. Med. Mol. Imaging.

[59] Benedetti F., Palladini M., Paolini M., Melloni E., Vai B., De Lorenzo R., Furlan R., Rovere-Querini P., Falini A., Mazza M.G. (2021). Brain correlates of depression, post-traumatic distress, and inflammatory biomarkers in COVID-19 survivors: A multimodal magnetic resonance imaging study. Brain Behav. Immun. Health.

[60] Xie Y., Xu E., Al-Aly Z. (2022). Risks of mental health outcomes in people with covid-19: Cohort study. BMJ.

[61] Sy M., Kitazawa M., Medeiros R., Whitman L., Cheng D., Lane T.E., Laferla F.M. (2011). Inflammation induced by infection potentiates tau pathological features in transgenic mice. Am. J. Pathol..

[62] De Chiara G., Piacentini R., Fabiani M., Mastrodonato A., Marcocci M.E., Limongi D., Napoletani G., Protto V., Coluccio P., Celestino I. (2019). Recurrent herpes simplex virus-1 infection induces hallmarks of neurodegeneration and cognitive deficits in mice. PLoS Pathog..

[63] Engler-Chiurazzi E.B., Russell A.E., Povroznik J.M., McDonald K.O., Porter K.N., Wang D.S., Hammock J., Billig B.K., Felton C.C., Yilmaz A. (2023). Intermittent systemic exposure to lipopolysaccharide-induced inflammation disrupts hippocampal long-term potentiation and impairs cognition in aging male mice. Brain Behav. Immun..

