# Supplementary material for: Emerging Role of Kinin B1 Receptor in Persistent Neuroinflammation and Neuropsychiatric Symptoms in Mice Following Recovery from SARS-CoV-2 Infection
Source: Cells. 2023 Aug 19;12(16):2107. doi: 10.3390/cells12162107 (PMC10453171; doi:10.3390/cells12162107)
Supplement: Supplementary file 1 [file cells-12-02107-s001.zip › cells-2552651-supplementary.pdf]

SUPPLEMENTARY DATA

Emerging role of kinin B1 receptor in persistent neuroinflammation and neuropsychiatric symptoms in mice following recovery from SARS-CoV-2 infection

Srinivas Sriramula[1*], Drew Theobald[1], Rohan Umesh Parekh[1], Shaw M. Akula[2], Dorcas P. O'Rourke[3], and Jeffrey B. Eells[4*]

[1]Department of Pharmacology and Toxicology, [2]Department of Microbiology and Immunology, [3]Department of Comparative Medicine, [4]Department of Anatomy and Cell Biology, Brody School of Medicine at East Carolina University, Greenville, NC 27834, USA.

***Corresponding Authors:**

Dr. Srinivas Sriramula, PhD
Department of Pharmacology and Toxicology,
Brody School of Medicine, East Carolina University
600 Moye Blvd,
Greenville, NC 27834, USA.
Phone number: +1 252-744-2746
Fax number: +1 252-744-3203
E-mail: sriramulas17@ecu.edu

Dr. Jeffrey B. Eells, PhD
Department of Anatomy and Cell Biology,
Brody School of Medicine, East Carolina University
600 Moye Blvd,
Greenville, NC 27834, USA.
Phone number: +1 252-744-2819
Fax number: +1 252-744-2850
E-mail: eellsj17@ecu.edu

Table S1. Primer sequences used for real-time quantitative reverse transcriptase polymerase chain reaction (qRT-PCR).

Gene	Forward primer (5'-3')	Reverse primer (5'-3')
IL-6	TCC TTA GCC ACT CCT TCT GT	AGC CAG AGT CCT TCA GAG A
TNF	TCT TTG AGA TCC ATG CCG TTG	AGA CCC TCA CAC TCA GAT CA
B1R	AGA TCA GAA GCT GCC AAG TTA G	TCC TGT CCT TCT TCC TTT TGC
AT1R	GGC TGG CAT TTT GTC TGG ATA	CAG TAG AAG AGT TAA GGG CCA T
β-Actin	GAC TCA TCG TAC TCC TGC TTG	GAT TAC TGC TCT GGC TCC TAG

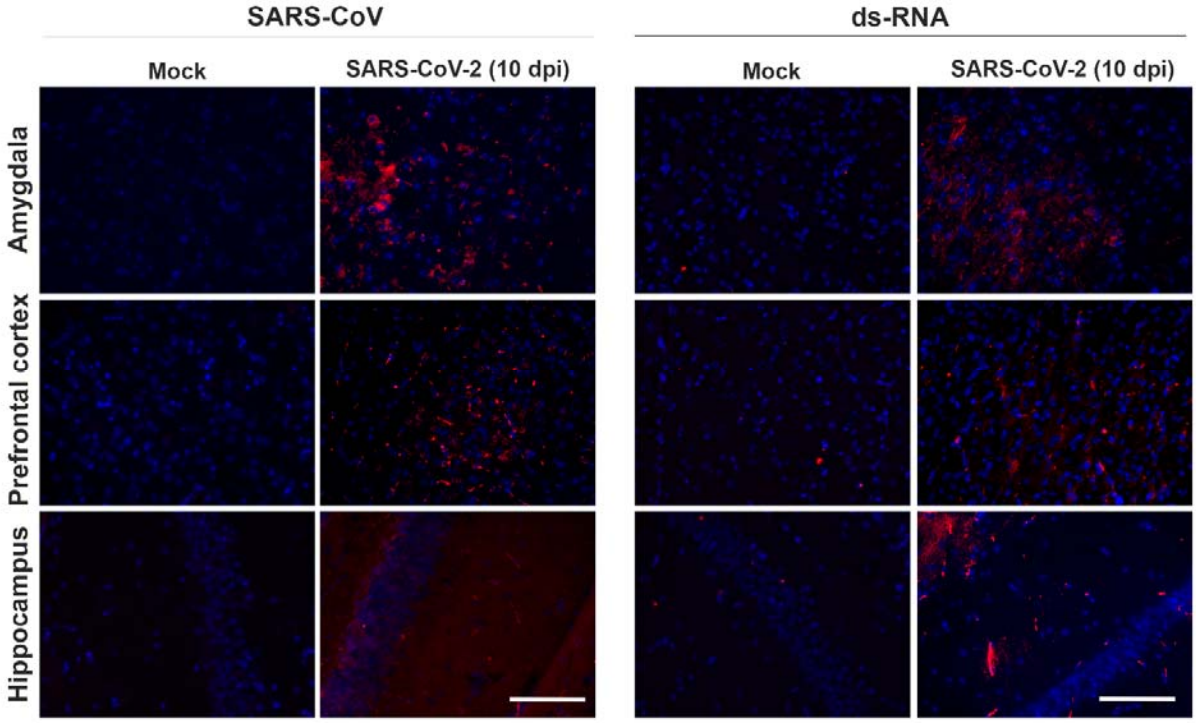

Figure S1. SARS-CoV-2 infected K18-hACE2 mice show presence of virus in the brain. Representative images showing immunofluorescence staining of SARS-CoV virus and double stranded RNA (dsRNA) in amygdala, prefrontal cortex, and hippocampus of mock or virus infected mice. Staining reveals that SARS-CoV virus is detectable at 10 dpi in all 3 regions of virus infected mice compared to mock infected. Detection of dsRNA in 10 dpi mice indicates active viral replication compared to mock infected mice. Scale bar: 100 μM.

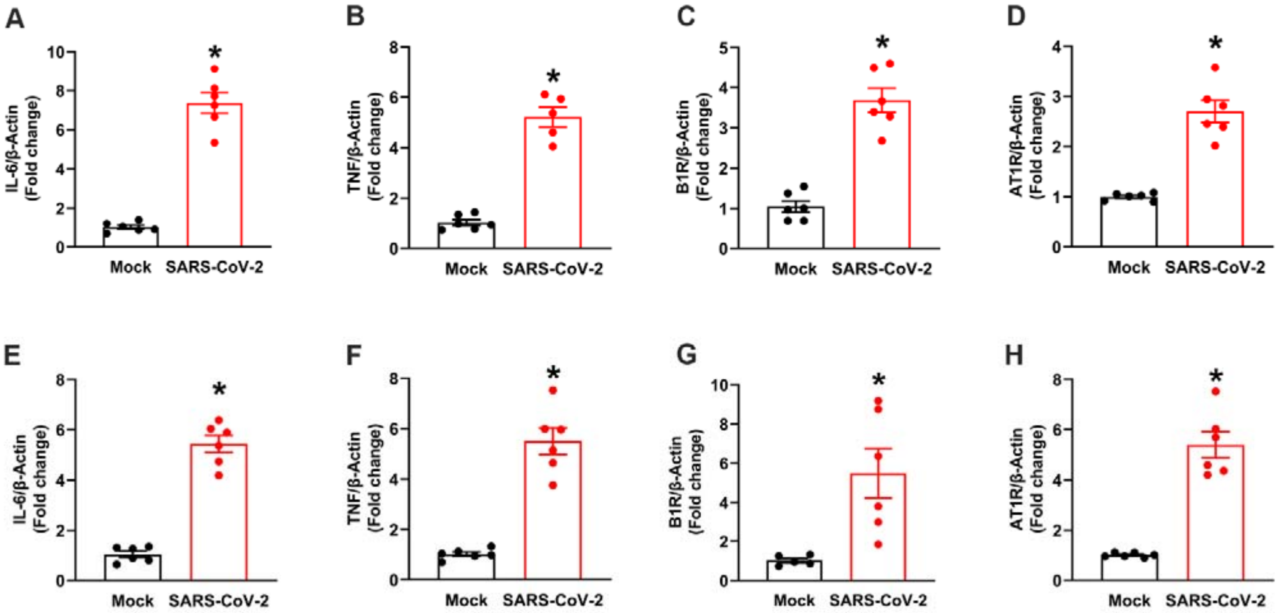

Figure S2. SARS-CoV-2 infection increases inflammatory markers in the brain.
Real time RT-PCR was used to measure mRNA expression of proinflammatory mediators in hippocampus and prefrontal cortex punches from mock and SARS-CoV-2 infected mice 45dpi. mRNA expression of IL-6, TNF, B1R, and AT1R is upregulated within the hippocampus **(A-D)** and prefrontal cortex **(E-H)** of SARS-CoV-2 infected mice compared to mock infected mice, indicating increased neuroinflammation. n=6 per group. Data are mean ± s.e.m. *p<0.05 (Student's t-test).

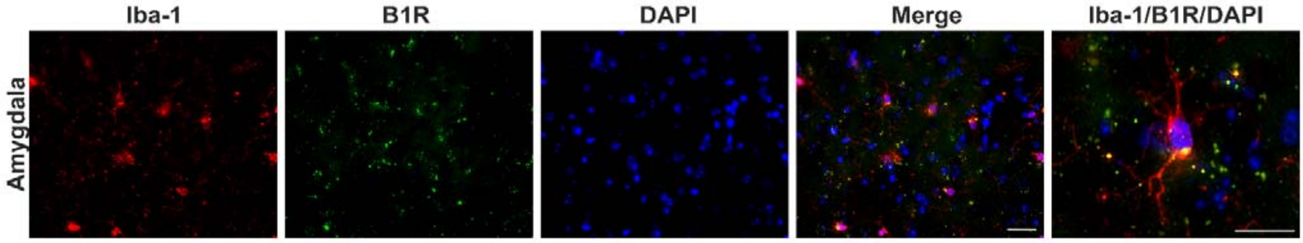

Figure S3. Co-localized expression of B1R with microglia.

Representative double immunofluorescence staining for microglia stained with ionized calcium-binding adaptor molecule 1 (Iba-1, red) and B1R expression (green) showing co-localization of B1R with microglia within the amygdala region of brain in SARS-CoV-2 infected mice. Scale bar: 100 uM.

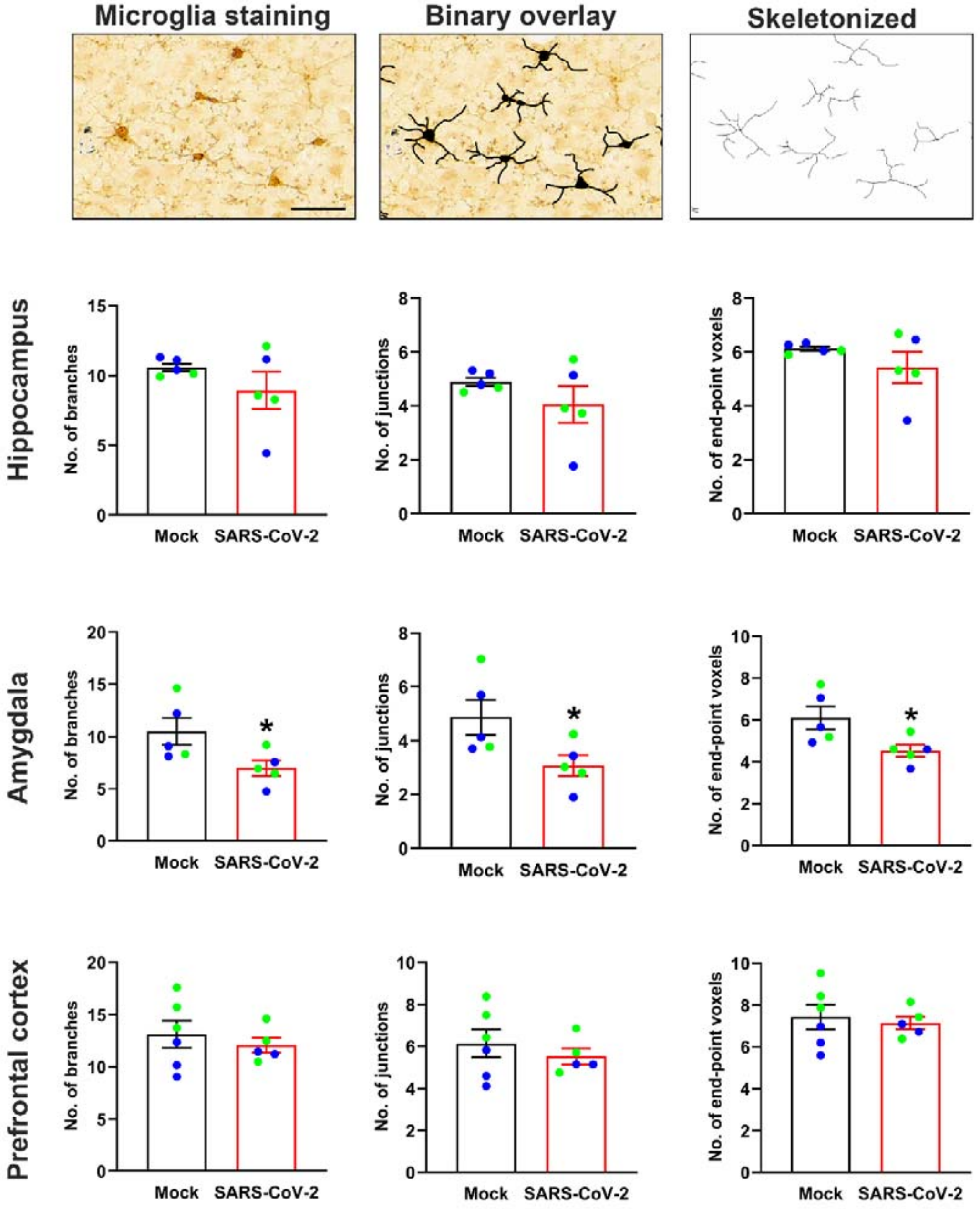

Figure S4. Mild infection with SARS-CoV-2 and microglia ramifications in different brain regions. Microglia morphology was quantified using photomicrographs of tissue in three different regions of the brain. The images were made binary, skeletonized, and analyzed using ImageJ plugins AnalyzeSkeleton (2D/3D) to determine the complexity of process branching (number of branches, number of junctions, and number of endpoints). Within the amygdala, there was a significant difference among the mock and SARS-CoV-2 infected groups for microglia in no. of branches, no. of junctions, and no. of end-point voxels. Blue data points represent male mice, green data points represent female mice. Data are mean ± s.e.m. *$p < 0.05$ (Student's t-test).
